# Supplementary material for: Structural Characteristics and Phylogenetic Analysis of the Mitochondrial Genomes of Four Krisna Species (Hemiptera: Cicadellidae: Iassinae)
Source: Genes (Basel). 2023 May 28;14(6):1175. doi: 10.3390/genes14061175 (PMC10297956; doi:10.3390/genes14061175)
Supplement: Supplementary file 1 [file genes-14-01175-s001.zip › Tables S1 and S2.pdf]

Table S1. The species used in phylogenetic analyses.

Family/Subfamily	Species	Accession Number
**Outgroups**	*Callitettix_braconoides*	NC_025497
	Magicicada_tredecim	NC_041652
	Tettigades_auropilosa	MG737767
**Treehopper**	*Maurya_qinlingensis*	NC_044706
	Tricentrus_brunneus	NC_044708
	Centrotus_cornutus	KX437728
	Leptocentrus_albolineatus	NC_044707
	Hypsauchenia_hardwickii	NC_044705
	Entylia_carinata	NC_033539
**Ledrinae**	*Tituria_pyramidata*	NC_046701
	Tituria_sagittata	NC_051528
	Petalocephala_gongshanensis	MW018818
	Petalocephala_chlorophana	NC_051527
	Petalocephala_eurglobata	MW018817
	Ledra_auditura	MK387845
**Evacanthinae**	*Evacanthus_danmainus*	MN227166
	Evacanthus_heimianus	MG813486
	Concaveplana_rufolineata	MN227168
**Typhlocybinae**	*Paraahimia_luodianensis*	NC047464
	Parathailocyba_orla	MN894531
	Parazyginella_tiani	MT683891
	Limassolla_lingchuanensis	NC046037
	Bolanusoides_shaanxiensis	MN661136
	Kusala_populi	NC064377
	Zyginella_minuta	MT488436
	Mitjaevia_protuberanta	NC047465
	Cassianeura_cassiae	NC062605
	Empoascanara_dwalata	MT350235
	Trifida_elongata	NC062822
	Empoasca_flavescens	MK211224
	Ghauriana_sinensis	MN699874
**Cicadellinae**	*Atkinsoniella_heiyuana*	MG813482
	Atkinsoniella_nigrita	NC064507
	Atkinsoniella_longiuscula	NC062846
	Bothrogonia_ferruginea	KU167550
	Bothrogonia_tongmaiana	NC049895
	Cicadella_viridis	MK335936
	Cofana_yasumatsui	NC049087
	Homalodisca_coagulata	AY875213
	Eupteryx_minuscula	MN910279
**Coelidiinae**	*Cladolidia_biungulata*	MW406474
	Cladolidia_robusta	MW406475

	Olidiana_ritcheri	NC057965
	Olidiana_ritcheriina	NC045207
	Taharana_fasciana	KY886913
	Olidiana_longisticka	NC057963
	Olidiana_obliquea	NC057964
	Olidiana_tongmaiensis	NC057966
**Iassinae**	*Krisna_concava*	NC046067
	Krisna_rufimarginata	NC046068
	Batracomorphus_lateprocessus	NC045858
	Trocnadella_arisana	NC036480
	Gessius_rufidorsus	MN577633
**Idiocerinae**	*Idiocerus_herrichii*	MN935487
	Idiocerus_salicis	MG813490
	Populicerus_confusus	NC050982
	Idioscopus_clypealis	MF784430
	Idioscopus_nitidulus	NC029203
**Macropsinae**	*Macropsis_notata*	NC042723
	Oncopsis_nigrofasciata	MG813492
**Megophthalminae**	*Durgades_nigropicta*	NC035684
	Japanagallia_spinosa	NC035685
**Deltocephalinae**	*Nephotettix_cincticeps*	NC026977
	Nephotettix_malayanus	NC066981
	Nephotettix_nigropictus	NC066982
	Nephotettix_virescens	NC066983
	Exitianus_indicus	KY039128
	Exitianus_nanus	NC066980
	Doratura_homophyla	NC066979
	Doratura_stylata	OK105077
	Maiestas_dorsalis	NC036296
	Alobaldia_tobae	KY039116
	Paralaevicephalus_gracilipenis	MK450366
	Yanocephalus_yanonis	NC036131
	Abrus_expansivus	NC045238
	Scaphoideus_maai	KY817243
	Scaphoideus_nigrivalveus	KY817244
	Scaphoideus_varius	KY817245
	Nesophrosyne_makaihe	NC066177
	Drabescoides_nuchalis	NC028154
	Nesophrosyne_maritima	NC066173
	Orosius_orientalis	KY039146
	Japananus_hyalinus	NC036298
	Macrosteles_quadrilineatus	NC034781
	Macrosteles_quadrimaculatus	NC039560
	Reticuluma_hamata	MN922303

**Hylicinae**	*Hylica_paradoxa*	NC056920
	Kalasha_nativa	NC056922
	Nacolus_tuberculatus	MW218663

Table S2. Correlation analysis between ENC, overall nucleotide and the corresponding nucleotide at the third codon position.

ATP6	T3%	C3%	G3%	GC3%	A3%
T%	0.731	0.67	0.266	0.54	-0.907
C%	0.068	0.515	-0.247	0.066	-0.103
A%	-0.631	-0.861	-0.396	-0.731	0.899
G%	-0.276	0.417	**0.981***	0.947	-0.146
GC%	-0.128	0.733	0.452	0.702	-0.19
ENC	0.929	0.435	-0.711	-0.316	-0.731
ATP8	T3%	C3%	G3%	GC3%	A3%
T%	0.245	-0.536	0.245	-0.486	0.346
C%	-0.917	0.464	-0.917	-0.42	0.698
A%	0	0.537	0	0.779	-0.693
G%	0.943	-0.904	0.943	-0.187	-0.167
GC%	-0.556	-0.12	-0.556	-0.837	0.942
ENC	-0.294	-0.21	-0.294	-0.655	0.687
COI	T3%	C3%	G3%	GC3%	A3%
T%	**0.998****	-0.38	-0.22	-0.346	-0.629
C%	-0.505	0.928	0.89	**0.971***	-0.317
A%	-0.611	-0.452	-0.639	-0.547	**0.991****
G%	0.459	0.544	0.782	0.664	-0.946
GC%	-0.119	0.877	**0.963***	**0.962***	-0.66
ENC	0.354	0.735	0.554	0.714	-0.89
COII	T3%	C3%	G3%	GC3%	A3%
T%	0.581	-0.288	0.533	-0.132	-0.573
C%	0.101	0.676	-0.853	0.543	-0.614
A%	-0.941	0.325	-0.304	0.303	0.847
G%	0.45	**-0.991****	0.897	**-0.976***	0.35
GC%	0.69	-0.235	-0.121	-0.401	-0.459
ENC	0.767	0.096	-0.131	0.082	**-0.989***
COIII	T3%	C3%	G3%	GC3%	A3%
T%	0.271	0.472	-0.03	0.307	-0.786
C%	-0.613	0.689	0.058	0.503	0.476
A%	0.295	-0.905	-0.267	-0.748	0.306
G%	-0.888	0.301	0.922	0.652	0.826
GC%	-0.898	0.686	0.471	0.7	0.762
ENC	-0.42	0.778	-0.083	0.495	0.155
CYTB	T3%	C3%	G3%	GC3%	A3%
T%	0.933	**-0.953***	0.656	-0.698	-0.891

C%	-0.299	0.678	-0.919	0.152	0.307
A%	**-0.988***	0.869	-0.41	0.779	0.931
G%	0.551	-0.874	0.872	-0.434	-0.52
GC%	-0.186	0.575	-0.912	0.028	0.212
ND1	T3%	C3%	G3%	GC3%	A3%
T%	0.845	-0.834	0.08	-0.436	-0.514
C%	-0.204	0.535	0.669	0.919	-0.245
A%	**-0.992****	0.736	-0.495	0.008	0.832
G%	**1.000****	-0.654	0.574	0.112	-0.893
GC%	0.867	-0.36	0.897	0.578	**-0.992****
ENC	-0.789	0.056	-0.705	-0.593	0.933
ND2	T3%	C3%	G3%	GC3%	A3%
T%	0.786	**-0.977***	0.565	0.187	-0.615
A3%	**-0.970***	0.607	**-0.990***	-0.888	1
C%	-0.448	0.827	-0.386	-0.054	0.329
A%	-0.926	0.836	-0.75	-0.479	0.82
GC%	0.862	-0.445	0.788	0.72	-0.865
ENC	0.899	-0.577	0.783	0.648	-0.864
ND3	T3%	C3%	G3%	GC3%	A3%
T%	**0.987***	-0.841	0.596	-0.622	-0.424
C%	-0.58	0.925	0.164	**0.958***	-0.311
A%	-0.495	-0.132	**-0.962***	-0.451	0.935
G%	**0.987***	-0.841	0.596	-0.622	-0.424
GC%	-0.399	0.847	0.365	0.949	-0.488
ENC	0.514	0.056	**0.952***	0.373	-0.881
ND4	T3%	C3%	G3%	GC3%	A3%
T%	**0.981***	-0.005	0.811	0.764	**-0.984***
C%	0.11	0.753	0.42	0.783	-0.293
A%	-0.947	-0.11	-0.862	-0.871	**0.985***
G%	0.704	0.127	0.948	**0.960***	-0.816
GC%	0.39	0.551	0.687	0.931	-0.556
ENC	-0.791	-0.013	**-0.983***	-0.936	0.879
ND4L	T3%	C3%	G3%	GC3%	A3%
T%	-0.828	0.676	**0.953***	0.9	-0.779
C%	0.3	0.233	0.09	0.115	-0.261
A%	0.863	-0.471	-0.844	-0.757	0.6
G%	**-0.979***	0.082	0.59	0.466	-0.205
GC%	-0.876	0.266	0.713	0.601	-0.411
ENC	-0.283	**0.997****	0.836	0.911	**-0.979***
ND5	T3%	C3%	G3%	GC3%	A3%
T%	0.917	-0.258	0.406	0.242	-0.903
C%	0.533	0.365	**0.996****	0.942	-0.698
A%	**-0.955***	0.201	-0.64	-0.454	**0.984***
G%	-0.306	0.152	0.365	0.354	0.207

GC%	-0.118	0.238	0.605	0.58	-0.016
ENC	**-0.968***	0.547	-0.281	-0.042	0.906
ND6	T3%	C3%	G3%	GC3%	A3%
T%	**0.951***	-0.787	-0.293	-0.713	-0.575
C%	-0.73	**0.975***	0.373	0.899	0.147
A%	**-0.975***	0.673	0.216	0.595	0.703
G%	0.696	**-0.956***	-0.261	-0.848	-0.144
GC%	-0.748	**0.981***	0.462	0.932	0.144
ENC	0.907	-0.273	-0.01	-0.203	-0.946

Bold**p < 0.01, *p < 0.05.

ATP6	T3%	C3%	G3%	GC3%	A3%
T%	0.731	0.67	0.266	0.54	-0.907
C%	0.068	0.515	-0.247	0.066	-0.103
A%	-0.631	-0.861	-0.396	-0.731	0.899
G%	-0.276	0.417	**0.981***	0.947	-0.146
GC%	-0.128	0.733	0.452	0.702	-0.19
ENC	0.929	0.435	-0.711	-0.316	-0.731
ATP8	T3%	C3%	G3%	GC3%	A3%
T%	0.245	-0.536	0.245	-0.486	0.346
C%	-0.917	0.464	-0.917	-0.42	0.698
A%	0	0.537	0	0.779	-0.693
G%	0.943	-0.904	0.943	-0.187	-0.167
GC%	-0.556	-0.12	-0.556	-0.837	0.942
ENC	-0.294	-0.21	-0.294	-0.655	0.687
COI	T3%	C3%	G3%	GC3%	A3%
T%	**0.998****	-0.38	-0.22	-0.346	-0.629
C%	-0.505	0.928	0.89	**0.971***	-0.317
A%	-0.611	-0.452	-0.639	-0.547	**0.991****
G%	0.459	0.544	0.782	0.664	-0.946
GC%	-0.119	0.877	**0.963***	**0.962***	-0.66
ENC	0.354	0.735	0.554	0.714	-0.89
COII	T3%	C3%	G3%	GC3%	A3%
T%	0.581	-0.288	0.533	-0.132	-0.573
C%	0.101	0.676	-0.853	0.543	-0.614
A%	-0.941	0.325	-0.304	0.303	0.847
G%	0.45	**-0.991****	0.897	**-0.976***	0.35
GC%	0.69	-0.235	-0.121	-0.401	-0.459
ENC	0.767	0.096	-0.131	0.082	**-0.989***
COIII	T3%	C3%	G3%	GC3%	A3%
T%	0.271	0.472	-0.03	0.307	-0.786
C%	-0.613	0.689	0.058	0.503	0.476
A%	0.295	-0.905	-0.267	-0.748	0.306
G%	-0.888	0.301	0.922	0.652	0.826
GC%	-0.898	0.686	0.471	0.7	0.762
ENC	-0.42	0.778	-0.083	0.495	0.155
CYTB	T3%	C3%	G3%	GC3%	A3%
T%	0.933	**-0.953***	0.656	-0.698	-0.891
C%	-0.299	0.678	-0.919	0.152	0.307

A%	**-0.988***	0.869	-0.41	0.779	0.931
G%	0.551	-0.874	0.872	-0.434	-0.52
GC%	-0.186	0.575	-0.912	0.028	0.212
ENC	0.888	-0.76	0.784	-0.357	-0.94
ND1	T3%	C3%	G3%	GC3%	A3%
T%	0.845	-0.834	0.08	-0.436	-0.514
C%	-0.204	0.535	0.669	0.919	-0.245
A%	**-0.992****	0.736	-0.495	0.008	0.832
G%	**1.000****	-0.654	0.574	0.112	-0.893
GC%	0.867	-0.36	0.897	0.578	**-0.992****
ENC	-0.789	0.056	-0.705	-0.593	0.933
ND2	T3%	C3%	G3%	GC3%	A3%
T%	0.786	**-0.977***	0.565	0.187	-0.615
A3%	**-0.970***	0.607	**-0.990***	-0.888	1
C%	-0.448	0.827	-0.386	-0.054	0.329
A%	-0.926	0.836	-0.75	-0.479	0.82
GC%	0.862	-0.445	0.788	0.72	-0.865
ENC	0.899	-0.577	0.783	0.648	-0.864
ND3	T3%	C3%	G3%	GC3%	A3%
T%	**0.987***	-0.841	0.596	-0.622	-0.424
C%	-0.58	0.925	0.164	**0.958***	-0.311
A%	-0.495	-0.132	**-0.962***	-0.451	0.935
G%	**0.987***	-0.841	0.596	-0.622	-0.424
GC%	-0.399	0.847	0.365	0.949	-0.488
ENC	0.514	0.056	**0.952***	0.373	-0.881
ND4	T3%	C3%	G3%	GC3%	A3%
T%	**0.981***	-0.005	0.811	0.764	**-0.984***
C%	0.11	0.753	0.42	0.783	-0.293
A%	-0.947	-0.11	-0.862	-0.871	**0.985***
G%	0.704	0.127	0.948	**0.960***	-0.816
GC%	0.39	0.551	0.687	0.931	-0.556
ENC	-0.791	-0.013	**-0.983***	-0.936	0.879
ND4L	T3%	C3%	G3%	GC3%	A3%
T%	-0.828	0.676	**0.953***	0.9	-0.779
C%	0.3	0.233	0.09	0.115	-0.261
A%	0.863	-0.471	-0.844	-0.757	0.6

G%	**-0.979***	0.082	0.59	0.466	-0.205
GC%	-0.876	0.266	0.713	0.601	-0.411
ENC	-0.283	**0.997****	0.836	0.911	**-0.979***
ND5	T3%	C3%	G3%	GC3%	A3%
T%	0.917	-0.258	0.406	0.242	-0.903
C%	0.533	0.365	**0.996****	0.942	-0.698
A%	**-0.955***	0.201	-0.64	-0.454	**0.984***
G%	-0.306	0.152	0.365	0.354	0.207
GC%	-0.118	0.238	0.605	0.58	-0.016
ENC	**-0.968***	0.547	-0.281	-0.042	0.906
ND6	T3%	C3%	G3%	GC3%	A3%
T%	**0.951***	-0.787	-0.293	-0.713	-0.575
C%	-0.73	**0.975***	0.373	0.899	0.147
A%	**-0.975***	0.673	0.216	0.595	0.703
G%	0.696	**-0.956***	-0.261	-0.848	-0.144
GC%	-0.748	**0.981***	0.462	0.932	0.144
ENC	0.907	-0.273	-0.01	-0.203	-0.946

**Genes**	**G3/(G3+C3)**	**A3/(A3+T3)**
ATP6	0.48	0.57
ATP8	0.26	0.72
COI	0.26	0.55
COII	0.19	0.58
COIII	0.43	0.48
CYTB	0.13	0.63
ND1	0.87	0.24
ND2	0.33	0.52
ND3	0.23	0.60
ND4	0.68	0.31
ND4L	0.71	0.33
ND5	0.79	0.32
ND6	0.22	0.58
