# Supplementary figures and images for: Structural Characteristics and Phylogenetic Analysis of the Mitochondrial Genomes of Four Krisna Species (Hemiptera: Cicadellidae: Iassinae)
Source: Genes (Basel). 2023 May 28;14(6):1175. doi: 10.3390/genes14061175 (PMC10297956; doi:10.3390/genes14061175)

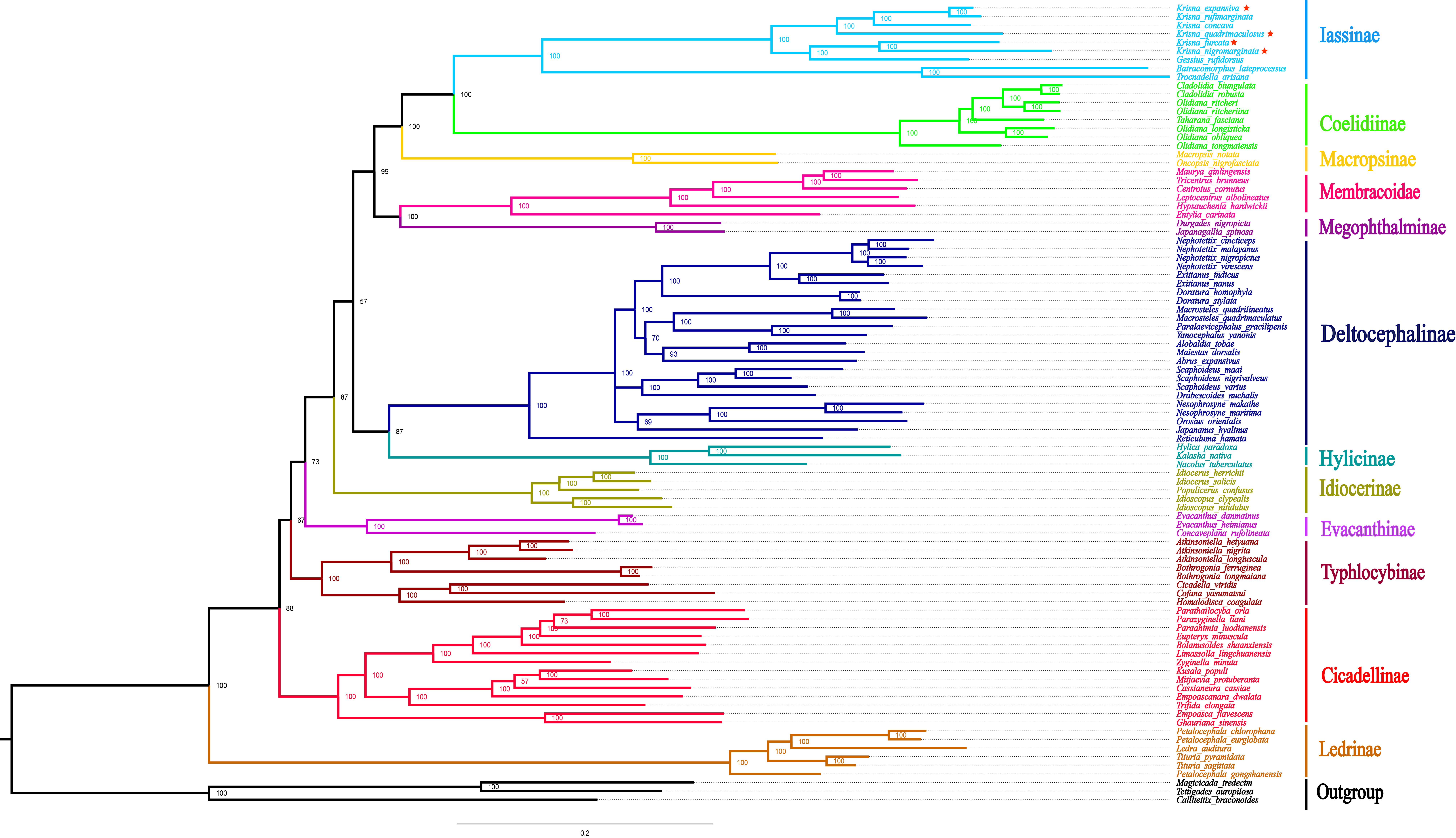

Supplement: Supplementary file 1 [file genes-14-01175-s001.zip › Figure S1 Phylogenetic tree of leafhoppers inferred by Bayesian inference based on nucleotides of PCG12RNA .jpg]

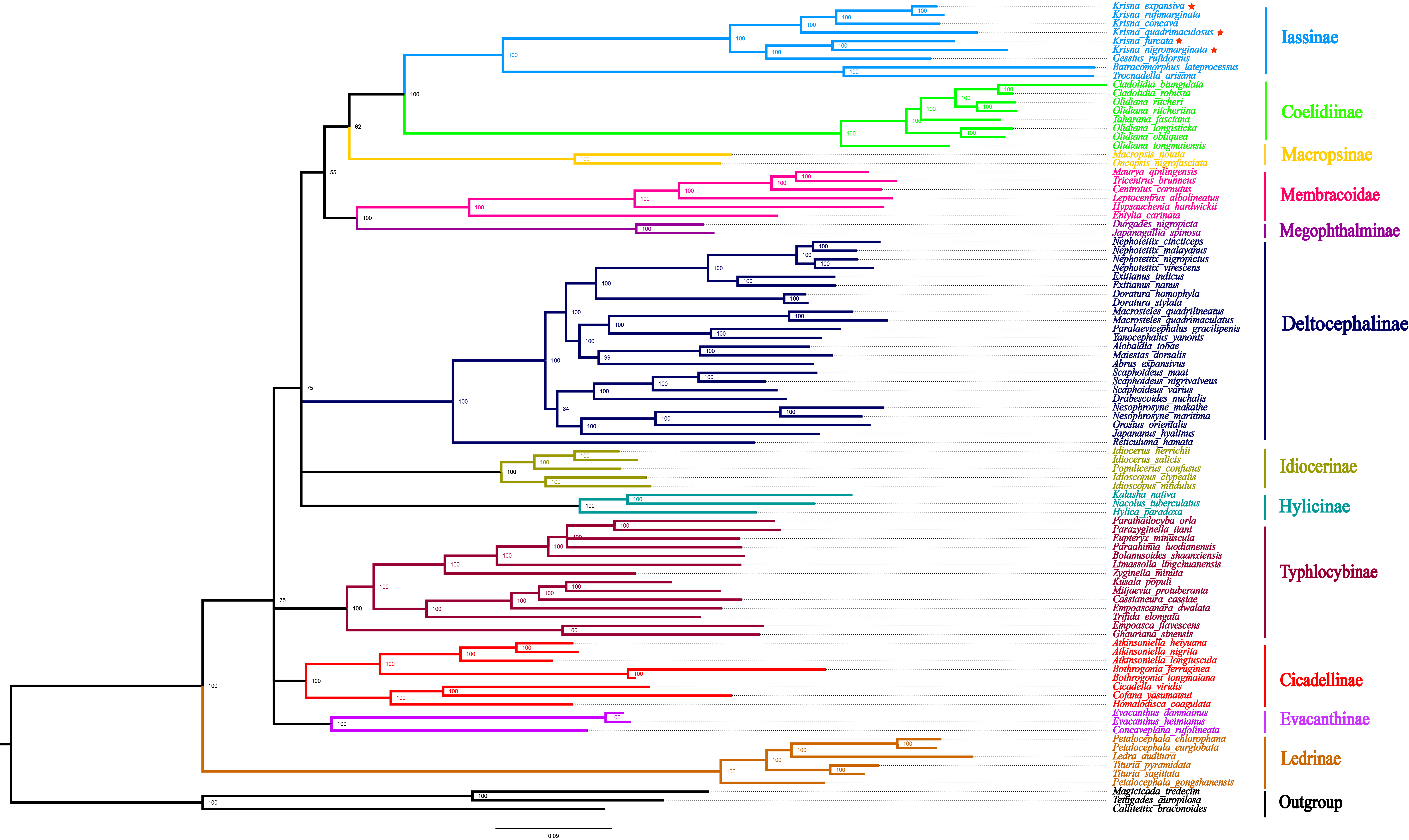

Supplement: Supplementary file 1 [file genes-14-01175-s001.zip › Figure S2 Phylogenetic tree of leafhoppers inferred by Bayesian inference based on nucleotides of PCG12 .jpg]

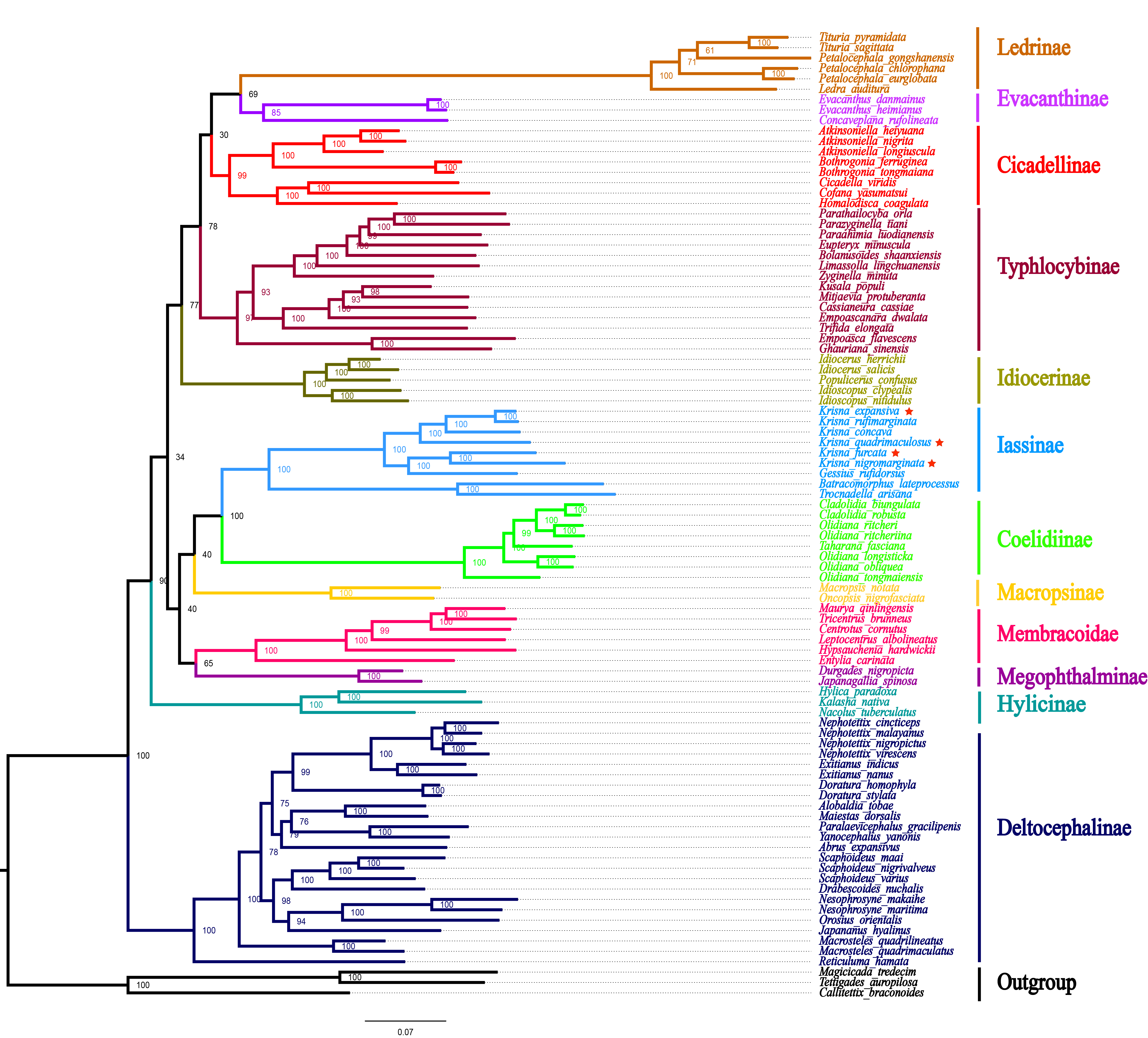

Supplement: Supplementary file 1 [file genes-14-01175-s001.zip › Figure S3 Phylogenetic tree of leafhoppers inferred by the maximum likelihood based on nucleotides of PCG12.jpg]
